# Systemic Blood Predictors of Elevated Pulmonary Artery Pressure Assessed by Non-invasive Echocardiography After Acute Exposure to High Altitude: A Prospective Cohort Study

**DOI:** 10.3389/fcvm.2022.866093

**Published:** 2022-06-10

**Authors:** Shi-Zhu Bian, Chen Zhang, Rong-Sheng Rao, Xiao-Han Ding, Lan Huang

**Affiliations:** ^1^Department of Cardiology, Xinqiao Hospital, Institute of Cardiovascular Diseases, Army Medical University (Third Military Medical University), Chongqing, China; ^2^Department of Ultrasonography, Xinqiao Hospital, Army Medical University (Third Military Medical University), Chongqing, China; ^3^Department of Health Care and Geriatrics, The 940th Hospital of Joint Logistics Support of Chinese People’s Liberation Army (PLA), Lanzhou, China

**Keywords:** predictors, elevated pulmonary artery pressure, acute high-altitude exposure, cohort study, vascular regulatory factors

## Abstract

**Aim:**

Elevated pulmonary artery pressure (ePAP) in response to high-altitude hypoxia is a critical physiopathological factor in the hypoxic adaptation that may lead to high-altitude pulmonary edema in the acute phase or high-altitude pulmonary hypertension in the long term. However, the sea-level predictors of risk factors for altitude-induced ePAP have not been examined. Thus, we aimed to identify the baseline systemic blood predictors of ePAP after acute high-altitude exposure.

**Materials and Methods:**

A total of 154 participants were transported to a high altitude 3,700 m from sea level within 2 h. Echocardiography examinations were performed to assess the mean pulmonary artery pressure (mPAP) and hemodynamics at both altitudes. All the individuals underwent blood tests to determine the concentrations of vascular regulatory factors. Univariate and adjusted logistic regression analyses were performed to identify the independent predictors of ePAP and factors related to ePAP.

**Results:**

The mPAP increased significantly from sea level to high altitude (19.79 ± 6.53–27.16 ± 7.16 mmHg, *p* < 0.05). Increased levels of endothelin (ET-1), Ang (1–7), Ang II, and bradykinin were found after high-altitude exposure, while the levels of nitric oxide (NO), prostaglandin E2 (PEG2), and serotonin decreased sharply (all *p*-values < 0.05). At high altitude, 52.6% of the subjects exhibited ePAP, and the mPAP was closely correlated with the baseline Ang II level (*r* = 0.170, *p* = 0.036) and follow-up levels of NO (*r* = −0.209, *p* = 0.009), Ang II (*r* = 0.246, *p* = 0.002), and Ang (1–7) (*r* = −0.222, *p* = 0.006) and the left atrial inner diameter (LAD, *r* = 0.270, *p* < 0.001). Both the baseline and follow-up NO and Ang II levels were significantly different between the ePAP and non-ePAP groups. Finally, we identified the baseline Ang II and NO concentrations as two independent predictors of ePAP (*p* < 0.05). We also found that two vascular regulatory factors with inverse roles, namely, Ang (1–7) and Ang II, at high altitudes were independently associated with ePAP. Additionally, ET-1, NO, PEG2, and LAD were associated with ePAP.

**Conclusion:**

The baseline concentrations of Ang II and NO at sea level are two independent predictors of ePAP after acute high-altitude exposure. Furthermore, Ang (1-7) and Ang II combined with ET-1, NO, PEG2, and LAD at high altitudes may contribute to the development of ePAP.

## Introduction

The physiological response of the pulmonary circulation to hypoxia is to increase pulmonary arteriolar resistance, resulting in elevated pulmonary artery pressure (ePAP) (1–6). Various degrees of increases in PAP at high altitudes have been reported in recent decades (3, 5–10). However, excessive ePAP in response to high-altitude hypoxia is a critical physiopathological factor in hypoxic adaptation leading to high-altitude pulmonary edema in the acute phase or high-altitude pulmonary hypertension (PH) in the long term (6, 11). In addition, the magnitude of hypoxic pulmonary vasoconstriction (HPV) in the high-altitude hypoxic environment is highly variable among humans, probably based on genetics and adaptive mechanisms (7, 12–14). Excessive HPV with ePAP may contribute to marked PH in sea-level residents with high-altitude exposure. Although a number of studies have investigated the prevalence and characteristics of high-altitude PH (HAPH) or isolated ePAP at high altitudes, few studies have reported the sea-level predictors of ePAP or risk factors for ePAP.

Previous studies assessed PAP in a limited number of populations by right heart catheterization (RHC), the recognized gold standard for PH at high altitudes (15, 16). However, at high altitudes, considering the reversibility of ePAP instead of HAPH in the acute phase after ameliorating hypoxia, invasive examination by RHC may be misused and be in contravention of the Declaration of Helsinki (2, 5, 6, 15, 16). Thus, it may be necessary to clarify the indications for RHC. As the technology for tools and tests has developed, rapid, non-invasive methods for evaluating ePAP have emerged, including echocardiography (15, 17). Echocardiography assessments of PAP have been widely used and have been recommended as the primary screening technology for PH at sea level by the American College of Cardiology Foundation (ACCF) (17). Non-invasive echocardiographic measurements are closely correlated with the gold standard (15). An echocardiography machine is also portable for high-altitude study and clinical practice.

In the past century, major studies in high-altitude medicine and physiology have focused on chronic high-altitude hypoxia-induced HAPH, which has been shown to be caused by hypoxia-induced pulmonary vascular remodeling (2, 5, 9). However, many reports have indicated an early phase onset of HAPH or increased PAP, even during only a few days of high-altitude exposure, which may be caused by hypoxia-induced pulmonary vasoconstriction (3, 6, 7). Although ePAP is not considered a type of acute mountain sickness and may recover to a normal level after a long period, it has been shown to limit activities of daily living (6). Additionally, ePAP has not been widely studied and is less understood, although it is reversible.

Numerous studies have focused on HAPH, and the alterations in PAP due to acute high-altitude exposure have not been thoroughly investigated (5, 8, 10, 14, 18). Acute high-altitude hypoxia-induced ePAP may be reversed after correcting hypoxia. During acute high-altitude exposure, PAP has been reported to significantly increase due to the hypoxic ventilatory response (HVR) and HPV (3, 4). We found that the increased mPAP decreased cardiopulmonary function and physical work capacity and even limited the activities of daily living of those unaccustomed to living at high altitudes (6). In recent decades, a reduction in the concentration of nitric oxide (NO) and an increase in the concentration of endothelin 1 (ET-1) have been shown to play pivotal roles in HAPH (19–21). Furthermore, prostaglandin E2 (PGE2) may also be involved in the development of HAPH, and two vascular regulatory factors with inverse roles, namely, Ang (1–7) and Ang II, have been shown to participate in systemic hypertension in low-altitude residents (22). However, their associations with PAP or HAPH are unknown, and the mechanisms underlying acute high-altitude hypoxic ePAP are not fully understood. There are also numerous other vascular regulatory factors that may contribute to the alterations in PAP due to high-altitude hypoxia that has not been studied in recent years.

Thus, we performed a field clinical trial based on a large population after acute high-altitude exposure to identify (1) the effect of acute high-altitude hypoxia exposure on PAP and (2) the baseline systemic blood risk factors for/predictors of increased PAP.

## Materials and Methods

### Participants

**FIGURE 1:**
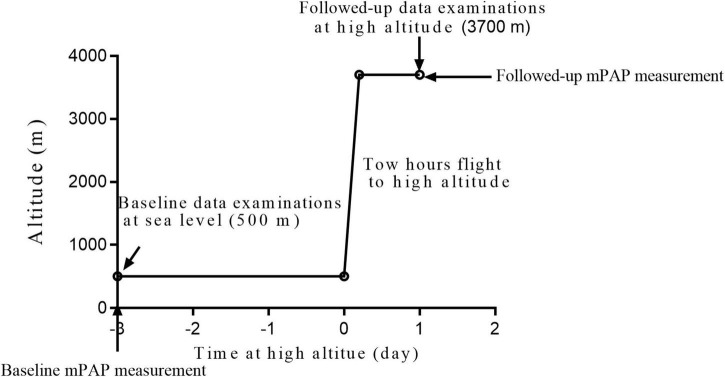
Ascent profile.

In total, 154 participants from Chengdu, Sichuan Province (sea level, average 500 m) and Lhasa (3,700 m above sea level) were recruited according to the inclusion and exclusion criteria (Figure 1).

The inclusion criteria were as follows: healthy men between 18 and 45 years of age. The exclusion criteria were as follows: individuals with hypertension, arrhythmia, myocarditis, or other cardiovascular diseases; primary headache; acute mountain sickness history; cold; pneumonia; pulmonary tuberculosis or other respiratory diseases; disorders of the liver or kidneys; malignant tumors; and neuropsychosis. In addition, people with high-altitude experience (above 2,500 m) within the past 2 years were excluded. The subjects were all young Chinese men with a similar age and body mass index (BMI). None of the subjects took medications regularly at low altitudes.

Smoking status was defined as smoking 1 or more cigarettes per day for at least 1 year. Alcohol users were defined as those drinking more than once a week (50 g white spirits or beer or red wine). Tea, coffee, or alcohol was avoided before the examinations. Our tests were conducted from 8:00 to 10:00 a.m.

The volunteers who agreed to participate in the trials were fully familiar with the purposes and procedures of the study and signed informed consent forms before the trials began. Our study was reviewed and approved by the Ethics Committee of Xinqiao Hospital at the Second Clinic Medical College of the Third Military Medical University and was carried out in accordance with established national and institutional ethical guidelines regarding the involvement of human subjects and the use of humans for research.

### Clinical Data Collection

The echocardiogram examinations were performed by our trained technician, Rong-Sheng Rao, with an ultrasonography system (CX50, Philips, United States). Further interpretations were recorded by a trained physician, Professor Shi-Yong Yu, from the Department of Cardiology. The end-diastolic internal diameters of the left atrium (LAD), left ventricle (LVDd), right atrium (RAD), right ventricle (RVD), pulmonary artery (PA), stroke volume (SV), and ejection fraction (LVEF) were measured. The heart rate (HR), tricuspid regurgitation area (TRA), and pulmonary artery acceleration time (PAT) were also recorded (Supplementary Figure 1).

Venous blood samples were obtained from the subjects between 8 and 10 a.m. after an overnight fast (at least 12 h). Plasma aliquots were obtained and stored at −80°C for further assays. Plasma ET-1, NO, PGE2, substance P (SP), bradykinin (BK), serotonin (5-HT), and Ang (1–7) and Ang II concentrations in the plasma from venous blood samples were measured using commercially available ELISA kits from Roche (Roche Diagnostics GmbH, Mannheim, Germany). All the biochemical variables were measured in the blood specimens at the Clinical Laboratory of Cardiology Science (Department of Cardiovascular Diseases) of Xinqiao Hospital, Army Medical University (Third Military Medical University), and Chinese People’s Liberation Army (PLA).

### Elevated Pulmonary Artery Pressure Definition

Since not all the subjects had tricuspid regurgitation, we assessed the mPAP using the PAT. The mPAP was calculated as follows according to previous reports: when the PAT was more than 120 ms, the mPAP was estimated using the formula mPAP = 79 − (0.45 × PAT). When the PAT was less than 120 ms, the mPAP was assessed using the formula mPAP = 90 − (0.62 × PAT). The ePAP group was defined as an mPAP greater than 25 mmHg according to the literature (15–17).

### Statistical Analysis

**FIGURE 2:**
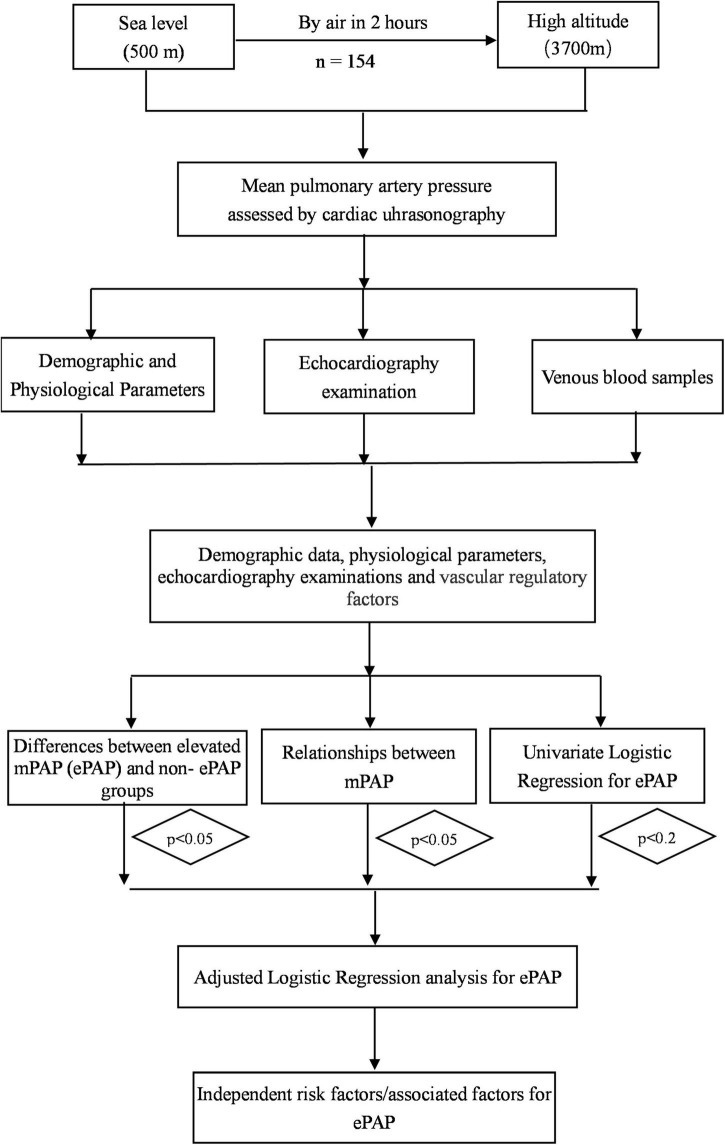
Flowchart of this study.

The data were excluded if the subjects’ demographic information and other items were incomplete. The normally distributed variables were expressed as means ± SD. The non-normally distributed variables were expressed as medians (Quartile 1 and 3). The ePAP group was defined as an mPAP greater than 25 mmHg according to the literature. The subjects have been divided into two groups (ePAP+ and ePAP− groups) according to the cutoff value of mPAP at high altitudes. Differences in the variables between the ePAP+ and ePAP− groups were assessed using independent sample *t*-tests or Mann–Whitney *U* tests as appropriate. The association between the variables and the mPAP was analyzed using Spearman correlation analysis. An adjusted logistic regression analysis was performed to identify independent risk factors for ePAP (i.e., those variables for which the *p*-value was less than 0.01 in the univariate analyses or *p* < 0.05 in the relationship analysis or *p* < 0.05 in the differences comparisons, Figure 2). A *p* ≤ 0.05 was considered statistically significant. The statistical analyses were performed using SPSS 22.0 for Windows (California, United States). All statistical methods and results were reviewed and approved by statisticians from the Army Medical University (Third Military Medical University).

## Results

### Basic Data of the Subjects

The mean age of the subjects was 22.58 ± 3.88 years, and the mean BMI was 21.77 ± 1.98 kg/m^2^. The incidence of ePAP in the 154 subjects was 52.6% (81/154) at high altitudes.

### Changes in Hemodynamics and Vascular Regulatory Factors

**FIGURE 3:**
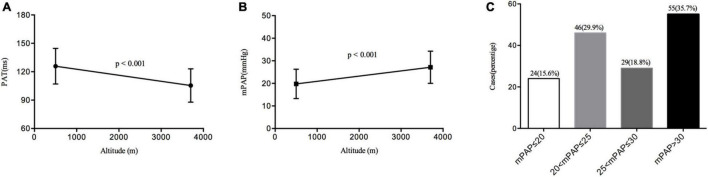
Changes in the pulmonary artery acceleration time (PAT) and mean pulmonary artery pressure (mPAP) distributions. **(A)** The change of PAT from sea level to high altitude. **(B)** The change of mPAP from sea level to high altitude. **(C)** The distribution of mPAP at high altitude.

In this study, the PAT was significantly shortened (125.89 ± 18.81 vs. 105.49 ± 17.68 ms, *p* < 0.001) by acute high-altitude hypoxia exposure. Consequently, the mPAP was markedly elevated from baseline (19.75 ± 6.53–27.16 ± 7.16 mmHg). Most of the subjects’ mPAP was in the normal range: 15.6% of the population had an mPAP ≤ 20 mmHg, 29.9% of the subjects had an mPAP greater than 20 and less than 25 mmHg, 18.8% of the population had an mPAP greater than 25 and less than 30 mmHg, and 35.7% of the subjects had an mPAP greater than 30 mmHg (Figure 3).

After acute high-altitude exposure, the hemodynamics showed a significant increase. In the hemodynamic parameters, the HR sharply increased from 68.61 ± 11.47 to 80.30 ± 12.10 bpm (*p* < 0.001). Furthermore, the EF also increased significantly, while the RV was significantly decreased (Table 1).

**TABLE 1:** Changes in hemodynamics and vascular regulatory factors after high-altitude exposure.

	Sea level	3,700 m	*p*-value
**Vascular regulatory factors**			
ET-1 (ng/ml)	0.50 (0.28–0.65)	0.62 (0.38–1.04)	<0.001
NO(μmol/L)	12.98 (11.52–15.47)	8.99 (6.76–9.99)	< 0.001
SP(pg/ml)	683.7 (612.08–720.63)	741.90 (601.07–942.84)	<0.001
PGE2 (pg/ml)	40.25 (37.21–41.87)	41.37 (40.11–44.79)	<0.001
BK(ng/ml)	8.36 (6.53–9.35)	20.78 (14.91–28.46)	<0.001
5-HT (ng/ml)	439.14 (347.91–4551.19)	368.84 (288.75–416.21)	<0.001
Ang (1–7) (pg/ml)	56.39 (44.37–74.67)	80.81 (64.05–97.38)	<0.001
Ang II (ng/ml)	0.60 (0.43–0.74)	0.79 (0.71–0.93)	<0.001
**Hemodynamic parameters**			
LAD (mm)	30.42 ± 1.99	29.29 ± 1.95	0.015
LVDd (mm)	46.16 ± 2.91	46.32 ± 2.53	0.539
LVEF (%)	63.81 ± 6.24	66.99 ± 4.47	<0.001
SV(ml)	64.99 ± 12.03	63.92 ± 13.40	0.385
RAD (mm)	35.56 ± 2.67	35.26 ± 3.93	0.374
RVD (mm)	34.96 ± 2.77	33.90 ± 2.67	<0.001
TRA (cm^2^)	0.50 (0.30–1.00)	0.50 (0.50–0.99)	0.790
TRV (cm/s)	211.00 (195.00–233.00)	247.00 (227.50–262.63)	<0.001
**Baseline vital parameters**			
HR (bpm)	68.10 ± 11.40	80.56 ± 12.12	<0.001
SBP (mmHg)	116.70 ± 11.20	116.97 ± 11.37	0.821
DBP (mmHg)	75.13 ± 9.73	77.42 ± 9.42	0.026
SpO_2_ (%)	98.31 ± 1.01	88.74 ± 2.88	<0.001

*The concentrations of vasoconstrictors increased significantly, while the concentrations of vasodilators decreased significantly after acute high-altitude exposure. The hemodynamics increased significantly from sea level to 3,700 m. BK, bradykinin; CI, confidence interval; LVEF, left ventricle ejection fraction; ePAP, elevated pulmonary artery pressure; ET-1, endothelin; HR, heart rate; mPAP, mean pulmonary artery pressure; LAD, left atrial inner diameter; LVDd, left ventricle diastolic diameter; NO, nitric oxide; OR, odds ratio; PA, pulmonary artery diameter; PAP, pulmonary artery pressure; PAT, pulmonary artery acceleration time; PEG2, prostaglandin E2; RAD, right atrium diameter; RVD, right ventricle diameter; SP, substance P; SV, stroke volume; TRA, tricuspid regurgitation area; TRV, tricuspid regurgitation velocity; 5-HT, serotonin.*

Regarding the vascular regulatory factors, the vasoconstrictor levels increased significantly. However, not all the vasodilator levels decreased significantly. The level of the strongest vascular constrictor, ET-1, increased from 0.50 (0.28–0.65) to 0.62 (0.38–1.04) ng/ml after acute hypoxia exposure (*p* < 0.001). The level of the strongest vascular dilator, NO, decreased significantly from 12.98 (11.52–15.47) to 8.99 (6.76–9.99) μmol/L (*p* < 0.001). Furthermore, the levels of 5-HT and PGE2 were also significantly reduced (both *p*-values less than 0.05). However, the level of BK increased significantly (*p* < 0.001, Table 1). The levels of both Ang (1–7) [56.39 (44.37–74.67) vs. 80.81 (64.05–97.38), *p* < 0.001] and Ang II [0.60 (0.43–0.74) vs. 0.79 (0.71–0.93), *p* < 0.001] significantly increased after acute high-altitude exposure for 24 h (*p* < 0.001, Table 1).

### Differences in Hemodynamics and Vascular Regulatory Factors Between the ePAP+ and ePAP− Groups

We compared the differences in hemodynamics and vascular regulatory factors between the ePAP+ and ePAP− groups. We did not find differences in the baseline (at sea level) hemodynamics, demographic data, or baseline vital parameters between the two groups. The baseline level of Ang II was significantly higher in the ePAP group [0.63 (0.47–0.77)] than in the non-ePAP group [0.56 (0.37–0.69), *p* = 0.002]. Furthermore, the baseline NO level was lower in the ePAP group [12.35 (10.63–14.61) vs. 13.46 (12.02–16.68) μmol/L, *p* = 0.031].

At follow-up, however, after acute exposure, the NO concentration was significantly lower in the ePAP+ group than in the ePAP− group (*p* = 0.46). We also found that Ang (1–7) [75.05 (57.18–91.28) vs. 85.15 (71.84–104.91) pg/ml, *p* = 0.034] and Ang II [0.82 (0.75–1.00) vs. 0.74 (0.68–0.87), *p* < 0.001] showed significant differences between the ePAP and non-PAP groups.

In the hemodynamic parameters at high altitude, the LAD was significantly higher in the ePAP+ group than in the ePAP− group (30.29 ± 1.80 vs. 29.48 ± 2.05 mm, *p* = 0.011). The other hemodynamic parameters and vascular regulatory factors did not show any differences between the groups at either altitude (Table 2).

**TABLE 2:** The differences in hemodynamics and vascular regulatory factors between the ePAP+ and ePAP− groups.

	Sea level			3,700 m		
	ePAP+ group (*n* = 81)	ePAP− group (*n* = 73)	*p* value	ePAP+ group (*n* = 81)	ePAP− group (*n* = 73)	*p*-value
**Demographic data**						
Age (year)	23.57 ± 4.50	22.80 ± 3.82	0.255	The same as sea level.		
BMI (kg/m^2^)	22.14 ± 2.50	21.57 ± 2.08	0.126	The same as sea level.		
**Vascular regulatory factors**						
ET-1 (ng/ml)	0.52 (0.31–0.65)	0.47 (0.25–0.65)	0.299	0.68 (0.38–1.22)	0.60 (0.38–1.00)	0.212
NO (μmol/L)	12.35 (10.63–14.61)	13.46 (12.02–16.68)	0.031	8.20 (6.66–9.99)	9.62 (6.75–11.91)	0.046
SP (pg/ml)	683.70 (608.79–719.78)	683.70 (622.55–724.37)	0.765	771.10 (584.74–976.48)	723.78 (608.28–913.37)	0.617
PGE2 (pg/ml)	40.27 (37.37–41.87)	39.81 (37.19–41.92)	0.775	41.28 (40.11–42.83)	41.59 (40.11–46.64)	0.103
BK (ng/ml)	8.36 (6.72–9.79)	8.08 (6.16–8.65)	0.055	20.78 (14.02–28.46)	20.78 (16.72–28.57)	0.560
5-HT (ng/ml)	439.14 (364.75–543.64)	439.14 (328.23–567.30)	0.949	368.47 (298.26–407.77)	371.26 (285.08–417.98)	0.714
Ang (1–7) (pg/ml)	59.12 (44.09–76.78)	52.81 (44.53–71.40)	0.749	75.05 (57.18–91.28)	85.15 (71.84–104.91)	0.034
Ang II (ng/ml)	0.63 (0.47–0.77)	0.56 (0.37–0.69)	0.002	0.82 (0.75–1.00)	0.74 (0.68–0.87)	< 0.001
**Hemodynamic parameters**						
LAD (mm)	30.47 ± 2.06	30.36 ± 1.93	0.721	30.29 ± 1.80	29.48 ± 2.05	0.011
LVDd (mm)	45.94 ± 2.88	46.41 ± 2.92	0.316	46.29 ± 2.47	46.34 ± 2.61	0.887
LVEF (%)	64.42 ± 4.92	63.13 ± 7.41	0.204	67.53 ± 4.95	66.40 ± 4.45	0.138
SV (ml)	65.65 ± 12.40	64.28 ± 11.64	0.475	64.42 ± 13.39	63.48 ± 13.70	0.702
RAD (mm)	35.34 ± 2.63	35.81 ± 2.71	0.278	35.53 ± 2.90	34.96 ± 4.84	0.372
RVD (mm)	34.58 ± 2.77	35.38 ± 2.73	0.073	34.16 ± 2.56	33.61 ± 2.78	0.207
TRA (cm^2^)	0.45 (0.20–0. 0.68)	0.52 (0.30–1.50)	0.012	0.50 (0.50–0.84)	0.60 (0.50–1.20)	0.669
TRV (cm/s)	204.00 (185.25–237.00)	213.00 (202.00–232.00)	0.248	250.00 (228.00–263.00)	246.00 (226.00–262.00)	0.528
**Baseline vital parameters**						
HR (bpm)	68.75 ± 11.87	67.37 ± 10.89	0.454	81.26 ± 12.38	79.79 ± 11.86	0.454
SBP (mmHg)	116.01 ± 9.96	117.47 ± 12.46	0.421	117.64 ± 12.00	116.24 ± 10.65	0.447
DBP (mmHg)	74.27 ± 9.42	76.10 ± 10.06	0.247	77.83 ± 9.44	76.96 ± 9.46	0.569
SpO_2_ (%)	98.24 ± 1.12	98.40 ± 0.88	0.334	89.07 ± 2.64	88.37 ± 3.11	0.136

*The differences in hemodynamics, vasoconstrictors, and vasodilators between the two groups at both altitudes.*

*ePAP, elevated pulmonary artery pressure.*

### Relationships Between the Mean Pulmonary Artery Pressure and the Hemodynamic and Vascular Regulatory Factors

We further analyzed the associations between the mPAP and the hemodynamics and vascular regulatory factors.

At baseline, only the Ang II level was closely positively related to the mPAP (*p* < 0.05). There was no significant association between the mPAP and the other vascular regulatory or hemodynamic factors.

**FIGURE 4:**
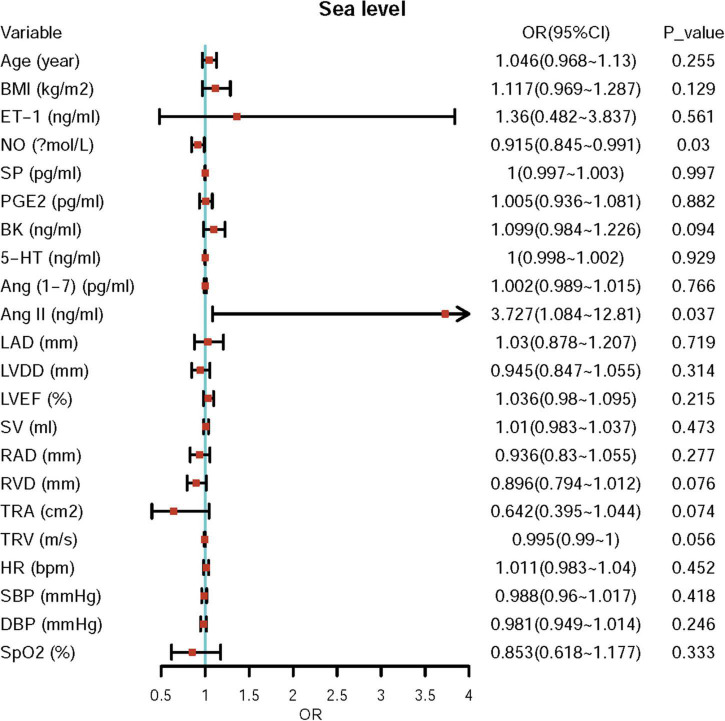
The potential predictor of elevated pulmonary artery pressure (ePAP) at sea level.

At 3,700 m, NO showed a strong relationship with the mPAP (*r* = −0.209, *p* = 0.009), while the LAD showed a negative relationship with the mPAP (*r* = −0.339, *p* < 0.0001). The Ang 1–7 and Ang II levels at high altitudes were also significantly correlated with the mPAP. The Ang 1–7 level was negatively associated with the mPAP (*r* = −0.222, *p* = 0.006), while the Ang II level was positively correlated with the mPAP (*r* = 0.246, *p* = 0.002). However, other hemodynamic parameters or vascular regulatory factors did not show close associations with the mPAP (Table 3 and Figure 4).

**TABLE 3:** Relationship between the mPAP and hemodynamics and vascular regulatory factors.

		Sea level	3,700 m	

	*r*	*p*	*r*	*p*
**Demographic data**				
Age (year)	0.130	0.109	The same as sea level.	
BMI (kg/m^2^)	0.144	0.075	The same as sea level.	
**Vascular regulatory factors**				
ET-1 (ng/ml)	0.122	0.131	0.064	0.426
NO (μmol/L)	–0.133	0.100	–0.209	0.009
SP (pg/ml)	–0.028	0.732	0.052	0.523
PGE2 (pg/ml)	0.001	0.998	–0.123	0.129
BK (ng/ml)	0.102	0.209	0.003	0.968
5-HT (ng/ml)	–0.046	0.567	0.013	0.871
Ang (1–7) (pg/ml)	0.031	0.706	–0.222	0.006
Ang II (ng/ml)	0.170	0.036	0.246	0.002
**Hemodynamic parameters**				
LAD (mm)	–0.004	0.956	0.270	<0.001
LVDd (mm)	–0.099	0.220	0.032	0.695
LVEF (%)	0.061	0.452	0.039	0.629
SV (ml)	–0.023	0.780	–0.014	0.862
RA (mm)	–0.107	0.187	0.012	0.879
RV (mm)	–0.141	0.081	0.089	0.272
TRA (cm^2^)	–0.020	0.832	–0.023	0.808
TRV (cm/s)	0.190	0.182	0.100	0.282
**Baseline vital parameters**				
HR (bpm)	0.0141	0.862	0.001	0.991
SBP (mmHg)	–0.091	0.266	0.086	0.287
DBP (mmHg)	–0.106	0.191	0.068	0.399
SpO_2_ (%)	–0.09	0.399	0.092	0.258

*Correlation analysis for the mPAP and other parameters at both altitudes.*

*mPAP, mean pulmonary artery pressure.*

### Logistic Regression Analyses of Independent Risk Factors/Predictors and Associated Factors

**FIGURE 5:**
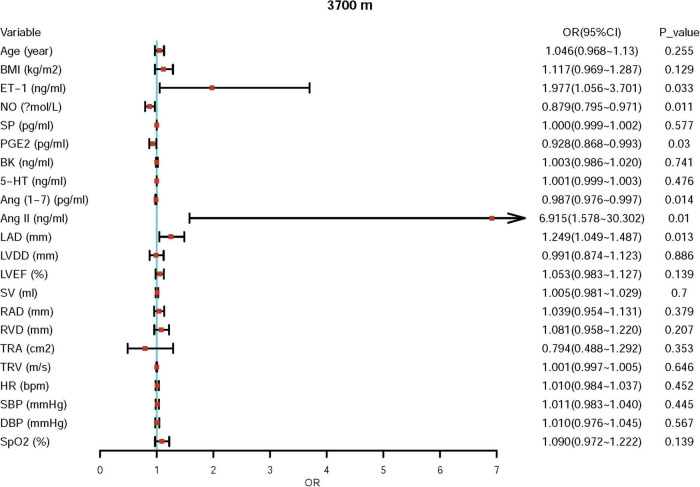
Univariate regression for ePAP at high altitude.

First, we performed univariate logistic regression using each variable at both sea level and high altitude. Univariate logistic regression showed that the baseline levels of NO, BK, RVD, TRA, TRV, and Ang II may be potential risk factors for ePAP or predictors of ePAP, and these factors were included in the adjusted regression. Based on the follow-up data, NO, ET-1, SP, and LAD may be associated with ePAP (Table 4 and Figures 4, 5).

**TABLE 4:** Univariate logistic regression for ePAP.

	Sea level					3,700 m				
	β	*p*	OR	95% CI		β	*p*	OR	95% CI	
**Demographic data**										
Age (year)	0.045	0.255	1.046	0.968	1.130	The same as sea level				
BMI (kg/m^2^)	0.110	0.129	1.117	0.969	1.287	The same as sea level				
**Vascular regulatory factors**										
ET-1 (ng/ml)	0.308	0.561	1.360	0.482	3.837	0.681	0.033	1.977	1.056	3.701
NO (μmol/L)	–0.088	0.030	0.915	0.845	0.991	–0.129	0.011	0.879	0.795	0.971
SP (pg/ml)	0.000	0.997	1.000	0.997	1.003	0.000	0.577	1.000	0.999	1.002
PGE2 (pg/ml)	0.005	0.882	1.005	0.936	1.081	–0.074	0.030	0.928	0.868	0.993
,BK (ng/ml)	0.094	0.094	1.099	0.984	1.226	0.003	0.741	1.003	0.986	1.020
5-HT (ng/ml)	0.000	0.929	1.000	0.998	1.002	0.001	0.476	1.001	0.999	1.003
Ang (1–7) (pg/ml)	0.002	0.766	1.002	0.989	1.015	–0.013	0.014	0.987	0.976	0.997
Ang II (ng/ml)	1.316	0.037	3.727	1.084	12.810	1.934	0.010	6.915	1.578	30.302
**Hemodynamic parameters**										
LAD (mm)	0.029	0.719	1.030	0.878	1.207	0.222	0.013	1.249	1.049	1.487
LVDD (mm)	–0.056	0.314	0.945	0.847	1.055	–0.009	0.886	0.991	0.874	1.123
LVEF (%)	0.035	0.215	1.036	0.980	1.095	0.052	0.139	1.053	0.983	1.127
SV (ml)	0.010	0.473	1.010	0.983	1.037	0.005	0.700	1.005	0.981	1.029
RAD (mm)	–0.067	0.277	0.936	0.830	1.055	0.038	0.379	1.039	0.954	1.131
RVD (mm)	–0.109	0.076	0.896	0.794	1.012	0.078	0.207	1.081	0.958	1.220
TRA (cm^2^)	–0.443	0.074	0.642	0.395	1.044	–0.231	0.353	0.794	0.488	1.292
TRV (m/s)	–0.005	0.056	0.995	0.990	1.000	0.001	0.646	1.001	0.997	1.005
**Baseline vital parameters**										
HR (bpm)	0.011	0.452	1.011	0.983	1.040	0.010	0.452	1.010	0.984	1.037
SBP (mmHg)	–0.012	0.418	0.988	0.960	1.017	0.011	0.445	1.011	0.983	1.040
DBP (mmHg)	–0.020	0.246	0.981	0.949	1.014	0.010	0.567	1.010	0.976	1.045
SpO_2_ (%)	–0.159	0.333	0.853	0.618	1.177	0.086	0.139	1.090	0.972	1.222

*Primary screening for the predictors of ePAP.*

*ePAP, elevated pulmonary artery pressure.*

**FIGURE 6:**
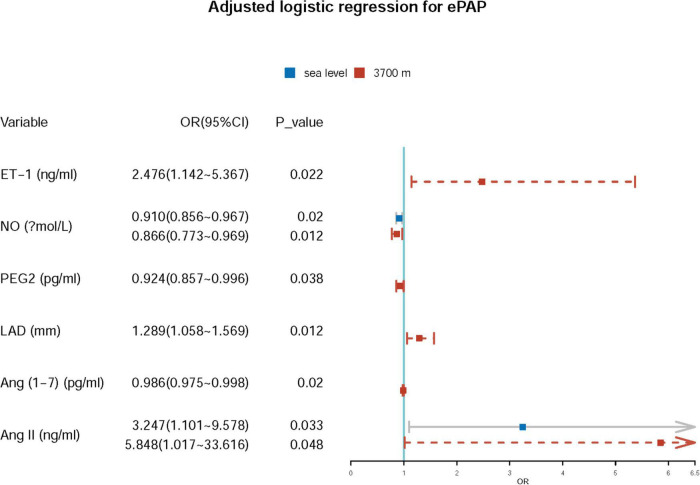
Adjusted regression for ePAP.

Further adjusted logistic regression indicated that lower NO (β = −0.094, *p* = 0.002; OR = 0.910; 95% CI: 0.856–0.967) and higher Ang II (β = 1.178; *p* = 0.033; OR = 3.247; 95% CI: 1.101–9.578) concentrations at baseline were two independent risk factors for predictors of ePAP. At high altitude, we identified several factors associated with ePAP, including ET-1, NO, PEG2, LAD, Ang (1–7), and Ang II (Table 5 and Figure 6).

**TABLE 5:** Adjusted logistic regression for ePAP.

	Sea level					3,700 m				
	β	*p*	OR	95% CI		β	*p*	OR	95% CI	
ET-1 (ng/ml)	Not selected					0.907	0.022	2.476	1.142	5.367
NO (μmol/L)	–0.094	0.002	0.910	0.856	0.967	–0.144	0.012	0.866	0.773	0.969
PEG2 (pg/ml)	Not selected					–0.069	0.038	0.924	0.857	0.996
LAD (mm)	Not selected					0.254	0.012	1.289	1.058	1.569
Ang (1–7) (pg/ml)	Not selected					–0.013	0.020	0.986	0.975	0.998
Ang II (ng/ml)	1.178	0.033	3.247	1.101	9.578	1.766	0.048	5.848	1.017	33.616

*Independent risk factors/predictors of ePAP.*

*ePAP, elevated pulmonary artery pressure.*

## Discussion

We first investigated the alterations in Ang (1–7) and Ang II from sea level to high altitude and identified that the baseline NO and Ang II concentrations are two independent predictors of ePAP. We found that the PAT decreased significantly after acute high-altitude exposure to 3,700 m within 24 h. Consequently, the mPAP increased significantly. We also found changes in hemodynamics and vascular regulatory factors. Furthermore, the sea-level baseline NO and Ang II concentrations are two independent predictors of ePAP after high-altitude exposure. Additionally, at high altitudes, we also found the associations of ET-1, NO, PEG2, Ang (1–7) and Ang II concentrations, and ePAP.

We found that the hemodynamics was enhanced by high-altitude exposure within 24 h. The HR increased significantly, which may be caused by hypoxia-induced sympathetic nerve activation (21, 23). However, the SV did not change significantly. These changes may be needed to supply sufficient blood and oxygen to the body under high-altitude hypoxic conditions. These alterations are compensatory responses to hypoxia. Furthermore, we discovered that in pulmonary hemodynamics, the PAT decreased sharply, while the mPAP increased significantly, which may also be compensatory responses. The enhanced hemodynamics was in accordance with previous studies and our reports.

The previous studies have indicated that systolic PAP calculated from tricuspid regurgitation and the mPAP calculated from the PAT are closely correlated (15, 17). However, their comparison has not been tested directly. In this study, we assessed PAP using the PAT instead of tricuspid regurgitation because not all the subjects suffered from tricuspid regurgitation.

Vascular regulation induced by hypoxia at high altitudes occurs by various mechanisms. First, vasoactive substances play critical roles in the vascular response to hypoxia. ET-1 levels showed a sharp increase within the first 24 h, while the strongest vasodilator, NO, displayed a dramatic reduction, which is consistent with previous studies (20, 21, 24). It is possible that hypoxia may constrict vessels by increasing the concentrations of vasoconstrictors and reducing the concentrations of vasodilators. However, regarding other regulatory factors, BK levels increased approximately 2-fold, while SP levels did not change significantly. These findings are similar to those of other reports on the effects of high-altitude exposure on BK and SP levels (25, 26). The changes in these vascular regulatory factors may be attributed to acute high-altitude stress.

It is well known that Ang II is one of the classic members of the renin-angiotensin-aldosterone system (RAAS), which participates in the development of hypertension. We found that Ang II levels rose significantly from sea level after acute high-altitude exposure, indicating that acute hypoxia may trigger the activation of the RAAS. Another study showed that angiotensin receptor blockers (ARBs) work with fewer effects at altitudes higher than 5,300 m, which may be attributed to the activation of the RAAS, including an increase in Ang II levels (27). Furthermore, ePAP populations have a higher level of Ang II both at sea level and high altitude (27).

In addition, we assessed another newly identified vascular regulator, Ang (1–7), which is produced from Ang I and Ang II. Ang (1–7) has been demonstrated to function in the dilation of blood vessels in an NO- and BK-dependent manner. Ang (1–7) may also play an inverse role to Ang II (27, 28). In contrast, both Ang (1–7) and Ang II are produced from Ang I; thus, the increase in Ang (1–7) may lead to a decrease in Ang II (29, 30). Thus, the balance between these factors after high-altitude hypoxia exposure may participate in the development of ePAP. This is the first report on the association between ePAP and Ang II or Ang (1–7).

Previous studies of hypoxia exposure have indicated that levels of constrictive factors increase significantly, while those of most vasodilators decrease significantly, resulting in vessel constriction, which causes an increase in blood pressure and increased velocities of blood flow and blood vessel resistance, including pulmonary arteries, thus leading to enhanced pulmonary hemodynamics (18, 23).

We further analyzed the associations between ePAP and hemodynamics. As discussed above, the hypoxia-induced constriction of blood vessels enhances hemodynamics. Although the HR increased significantly from sea level to high altitude in accordance with a previous study, neither the mPAP nor HAPH showed any associations with the HR. Furthermore, we did not find a correlation between ePAP and right heart function or left heart function. However, the mPAP was closely associated with the LAD, which may be caused by the interaction between the left heart and right heart (7).

In this study, we examined several commonly recognized vascular regulatory factors to identify the predictors of risk factors for ePAP. First, we investigated the strongest vasoconstrictor, ET-1, the concentration of which increased significantly after high-altitude hypoxia exposure. However, we did not find any associations or differences between mPAP or ePAP and the baseline ET-1 level. Previous studies have reported that ET-1 plays critical roles in PH and HAPH, as it is involved in the underlying mechanisms, including promoting the proliferation of both endothelial cells (ECs) and vascular smooth muscle cells (VSMCs) as well as the constriction of the pulmonary artery due to EC dysfunction (31–33). However, the findings of this study were inconsistent with the results of several other studies, possibly due to the limited sample size in combination with the high-altitude exposure pattern.

Several vasodilators were also assessed in this study, including SP (a type of tachykinin and inflammatory factor), PGE2 (permeability), and 5-HT (emotion regulation), which may be significantly changed by high-altitude hypoxia. After high-altitude stress, the circulatory concentrations of the abovementioned vasodilators changed significantly, except for SP. These results were partly in accordance with previous studies. However, we did not find any associations with ePAP in either the correlation analysis or logistic regression.

In addition, we investigated the strongest vasodilator, NO, which has been shown to be effective for treating HAPH as well as to have a close association with HAPH. However, the predictive role of the baseline NO level at sea level in ePAP after acute exposure is unknown. In accordance with a previous report, we found that after high-altitude exposure, both the baseline and high-altitude NO concentrations were significantly associated with the mPAP. Furthermore, the baseline NO concentration at sea level also showed significant differences between the ePAP− and ePAP+ groups. We also identified that a lower baseline NO level is an independent predictor of ePAP.

Finally, the newly identified vascular regulator Ang (1–7) and the hypertension contributor Ang II were also investigated (16, 20, 27, 34). The baseline level of Ang II showed a significant association with ePAP at both altitudes, indicating its role in ePAP subjects and their susceptibility. However, Ang (1–7) at baseline showed no association with ePAP. After high-altitude exposure, Ang (1–7) was significantly lower in the ePAP subjects, showing that Ang (1–7) may be a protective factor against ePAP.

This cohort study was designed to identify the baseline risk factors for ePAP or predictors of ePAP resulting from high-altitude exposure. After correlation analysis and univariate and adjusted logistic analyses, we found that a lower baseline NO level and a higher baseline level of Ang II were predictors of ePAP and had not been previously identified. A previous study reported a decrease in exhaled nitric oxide on chronic intermittent hypoxia exposure in well-acclimatized mine workers. The above-mentioned literature investigated the effect of chronic hypoxia on the respiratory tract which may play a role in the acclimatization (35). Another study has shown that acute exposure to hypobaric hypoxia in the mountains due to the shortage of oxygen as a substrate for NO synthesis leads to a decrease in exhaled NO in HAPE-susceptible individuals. The exhaled nitric oxide may be a valuable index of health or diseases at high altitudes (36). However, we did not measure the exhaled nitric oxide level on acute exposure to high altitude. This should be improved in our future studies and should be combined with the respiratory functions at high altitudes.

Additionally, at high altitudes, the levels of ET-1, NO, PEG2, Ang (1–7), and Ang II as well as the LA were independently associated with ePAP, which may indicate that they are involved in the physiopathological mechanisms of ePAP. Specifically, Ang (1–7) and Ang II or their balance may contribute to the development of ePAP. Ang (1–7) and Ang II play different roles in smooth muscle cell proliferation, EC function maintenance, and systemic hypertension (16, 20, 27, 34). Furthermore, considering their competitive relations, their interactions and their roles in the underlying mechanisms of ePAP/HAPH should be given sufficient attention in future studies. In addition, ePAP/HAPH may be a process of high-altitude illness, which affects the daily work or life among the population at high altitudes. Thus, the predictors of ePAP or characteristics for ePAP may provide the prevention for high altitude illness.

### Limitations

There are several limitations in our study. First, because not all the subjects suffered from TR, the mPAP was calculated using the PAT instead of TR or RHC. Second, the sample size was relatively small, which may have led to results that were inconsistent with those of other studies. Third, due to difficulties in conducting the field trial, we could only obtain the systemic vascular regulators, and it would be better to obtain the levels of local blood or tissue (such as pulmonary artery) vascular regulators to study their roles in ePAP. Fourth, the exposure altitude, which is a moderate altitude, was not high enough to induce a more significantly elevated PAP. Finally, in addition to our investigated factors, there are many other factors that we have not covered, which warrant further study.

## Conclusion

The mPAP was closely associated with Ang II and NO levels. Baseline Ang II and NO concentrations at sea level were two independent predictors of ePAP after acute high-altitude exposure with opposite effects. Furthermore, Ang (1–7) and Ang II at high altitudes may contribute to the development of ePAP after acute high-altitude exposure.

## Data Availability Statement

The original contributions presented in the study are included in the article/Supplementary Material, further inquiries can be directed to the corresponding author.

## Ethics Statement

The studies involving human participants were reviewed and approved by the Ethics Committee of Xinqiao Hospital at the Second Clinic Medical College of the Third Military Medical University. The patients/participants provided their written informed consent to participate in this study.

## Author Contributions

X-HD and LH participated in the design of this study. X-HD and S-ZB drafted the manuscript, performed the statistical analysis, and performed the analysis of PAP-related measurements. LH reviewed and revised the manuscript critically for important intellectual content. R-SR performed the echocardiography examination. CZ and S-ZB collected the demographic data and blood examination data. The other laboratory measurements were performed by X-HD, S-ZB, and CZ. All authors contributed to the article and approved the submitted version.

## Conflict of Interest

The authors declare that the research was conducted in the absence of any commercial or financial relationships that could be construed as a potential conflict of interest.

## Publisher’s Note

All claims expressed in this article are solely those of the authors and do not necessarily represent those of their affiliated organizations, or those of the publisher, the editors and the reviewers. Any product that may be evaluated in this article, or claim that may be made by its manufacturer, is not guaranteed or endorsed by the publisher.

## Supplementary Material

Supplementary Figure 1 Measurements of the pulmonary artery acceleration time (PAT) at sea level and high altitude.

## Abbreviations

BK: bradykinin
CI: confidence interval
LVEF: left ventricle ejection fraction
ePAP: elevated pulmonary artery pressure
ET-1: endothelin
HAPH: high-altitude pulmonary hypertension
HR: heart rate
mPAP: mean pulmonary artery pressure
LAD: left atrial inner diameter
LVDd: left ventricle diastolic diameter
NO: nitric oxide
OR: odds ratio
PA: pulmonary artery diameter
PAP: pulmonary artery pressure
PAT: pulmonary artery acceleration time
PEG2: prostaglandin E2
RAD: right atrium diameter
RVD: right ventricle diameter
SP: substance P
SV: stroke volume
TRA: tricuspid regurgitation area
TRV: tricuspid regurgitation velocity
5-HT: serotonin.
