# Supplementary figures and images for: Systemic Blood Predictors of Elevated Pulmonary Artery Pressure Assessed by Non-invasive Echocardiography After Acute Exposure to High Altitude: A Prospective Cohort Study
Source: Front Cardiovasc Med. 2022 Jun 10;9:866093. doi: 10.3389/fcvm.2022.866093 (PMC9226344; doi:10.3389/fcvm.2022.866093)

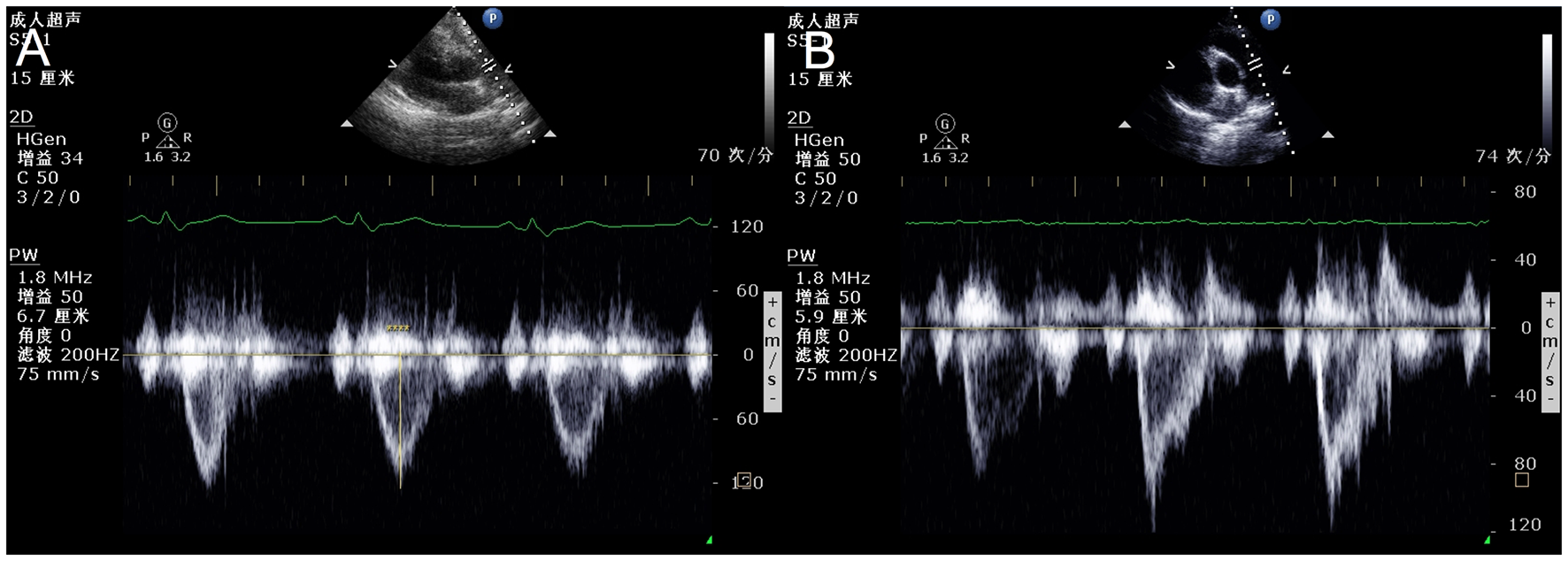

Supplement: Supplementary Figure 1 — Measurements of the pulmonary artery acceleration time (PAT) at sea level and high altitude. [file Image_1.TIFF]
